# Assembly and phylogenetic analysis of the complete mitogenome of *Devario interruptus* (Teleostei, Cypriniformes, Danionidae)

**DOI:** 10.1080/23802359.2022.2151831

**Published:** 2023-02-03

**Authors:** Xiao Jiang Chen, Wen Zhao Liu, Lin Song, Hai Xia Liu

**Affiliations:** a College of Fisheries Science and Technology, Jiangsu Agri-Animal Husbandry Vocational College, Taizhou City, Jiangsu Province, P. R. China; b College of Fisheries and Life Science, Dalian Ocean University, Dalian City, Liaoning Province, P. R. China

**Keywords:** Mitochondrial genome, *Devario interruptus*, *Barilius interrupta*, *Barilius interruptus*, *Brachydanio interrupta*

## Abstract

The complete mitochondrial DNA genome of *Devario* interruptus was sequenced on the Illumina HiSeq platform and found to be 16,735 bp and included 37 genes encoding 13 proteins, 22 tRNAs, two rRNAs, and two non-coding regions. The proportion of nucleotides in mitochondrial genome was T (27.9%), C (23.7%), A (33%), G (15.4%), and the deviation of AT was 60.9%. A Maximum-Likelihood phylogenetic tree was reconstructed using the concatenated mitochondrial protein-coding genes of *D. interruptus* and other 18 species of fishes. Phylogenetic analysis results supported that *D. interruptus* was closely related to *Devario shanensis*. Fundamental genetic data of *D. interruptus* will be essential for further genetic studies.

## 1. Introduction

**Figure 1.**
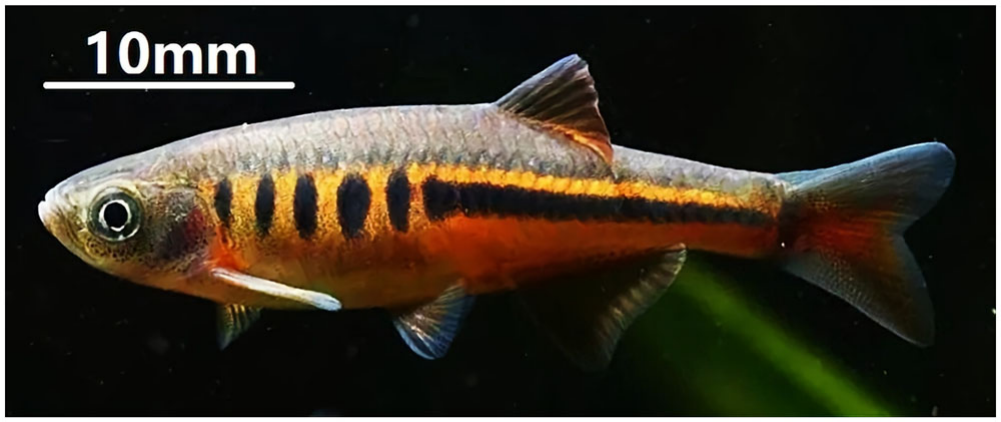
*S*pecimen of *Devario interruptus* was collected from the Yingjiang County, Yunnan Province, China. Photograph by Xiao Jiang CHEN on Jun 10, 2021.

*Devario interruptus* (Day, 1870), belonging to the subfamily Danioninae, family Danionidae, lives in the slow flow of mountain streams or ditches, and is distributed in Southeast Asian countries on both sides of the Irrawaddy River, especially on the north of Myanmar, and partially distributed in the Nujiang river system, Longchuan Jiang and Daying Jiang watersheds in Yunnan, China (Chu and Chen 1989). Barilius interrupta, Brachydanio interrupta, Barilius interruptus, and Danio interruptus are all formerly used taxonomic synonyms for *Devario interruptus*. The total length of *D. interruptus* ranges from 42 to 69 mm, and the species is characterized by dorsal fin 3,7; anal fin 2,10–12; pectoral fins 1,10–11; abdominal fin 1,6; longitudinal scales 34–36; transverse scales 9–10; dorsal fin anterior scale 16–18; gill rake 8–9; the head length is smaller than body height; the snout short with a depression in the center of the leading edge; eyes are more proximal to the rostral than to the posterior edge of the gill cover; the mandible is slightly prominent over the maxilla; the mouth cleft reaches up to the nares vertically inferiorly; small round scales cover the body and easy to fall off; the lateral line is incomplete; dorsal fins are free of rigid spines; 4–9 plaques on the body side from the posterior edge of the gill cap to the base of the dorsal fin, then a golden yellow longitudinal band extending to the base of the caudal fin; the ventral side of the male fish is slightly red, while the female fish is slightly yellow (Chu and Chen 1989; Fang 2000). The reference image was taken by Xiao Jiang CHEN on Jun 10, 2021 (Figure 1).

So far, research on *D. interruptus* is limited to morphological classification (Fang 2000; Fang 2003), and the lack of genetic information limits our current understanding of the evolutionary phylogenetic relationship of this species within family Danionidae. Therefore, it is of great significance to sequence the whole mitochondrial genome of *D. interruptus* as soon as possible. This project used high-throughput sequencing technology to carry out sequencing of the whole mitochondrial genome of *D. interruptus*.

## 2. Materials and methods

### 2.1. Sample collection and preservation

In this study, specimens of *D. interruptus* were collected from the Yingjiang County, Yunnan Province, China (24.493973° N, 97.737320° E) in Jun 2021, using small set nets and gill nets with the permit by Jiangsu Agri-animal Husbandry Vocational College (granted # NSF2021ZR14). To euthanize the fish, the experimental fish were euthanized using eugenol (0.2ml/l), and then transferred to 75% ethanol for 24 hours before being transitioned to 95% ethanol for prolonged storage. All specimens were deposited in the Aquatic Science and Technology Institution Herbarium (https://www.jsahvc.edu.cn/; Voucher number ASTIH-21b0616d02, Chen Xiao Jiang, 2007020030@jsahvc.edu.cn). According to the morphological characteristics of *D. interruptus* described by Chu and Chen 1989, the fish were observed, counted, and measured by using an anatomical microscope and other tools, and finally, the identification was completed.

### 2.2. DNA extraction, sequencing, and assembly

The tissue sample used for sequencing was kept together with the corresponding voucher samples, the Tguide Cell/tissue genomic DNA Extraction Kit (Tiangen, Beijing, China) was used to isolate DNA from the muscle tissue of a single adult specimen. The concentration of the DNA sample detected by NanoDrop 2000 (Thermo Fisher Scientific, USA). The sequencing library was prepared by random fragmentation of the DNA sample, followed by PCR amplification, size selection, and library quality check. The DNA raw reads were obtained from the sequencing of the constructed library on Illumina HiSeq 4000 Sequencing platform (Illumina, CA, USA). The quality check process was conducted on FastQC 0.11.8 (Andrews 2010), the main references are as follows conditions: (1) Sequences containing more than 3 N bases were eliminated; (2) High-quality bases (Phred score ≥20) accounting for less than 60% of sequences were removed; (3) Excluding 3′ end low-quality bases; (4) Sequences less than 60 bp in length were discarded. The sequences were assembled into contigs using metaSPAdes 3.13 (Nurk et al. 2017) with default parameters, and *Betadevario ramachandrani* MH817023 was take as reference (Norén & Kullander 2018), and then the resulting *D. interruptus* draft mitogenome assembly was further analyzed by comparison to the mitogenome of *B. ramachandrani* (Norén & Kullander 2018) both to confirm correct direction assembly of the contigs and identity the starting base position of the *D. interruptus* mitogenome.

### 2.3. Annotation and analysis

The resulting circular contig consensus sequence was annotated and verified by mitoMaker 1.14 and MITOS WebServer (http://mitos.bioinf.uni-leipzig.de/index.py) (Bernt et al. 2013). Genome Maps was generated using CGView Server (Grant and Stothard 2008; https://proksee.ca/). The alignments, analyses, model calculation, and phylogeny reconstruction were all completed by MEGA X (Kumar et al. 2018).

## 3. Results and discussion

### 3.1. Genomic characterization

**Figure 2.**
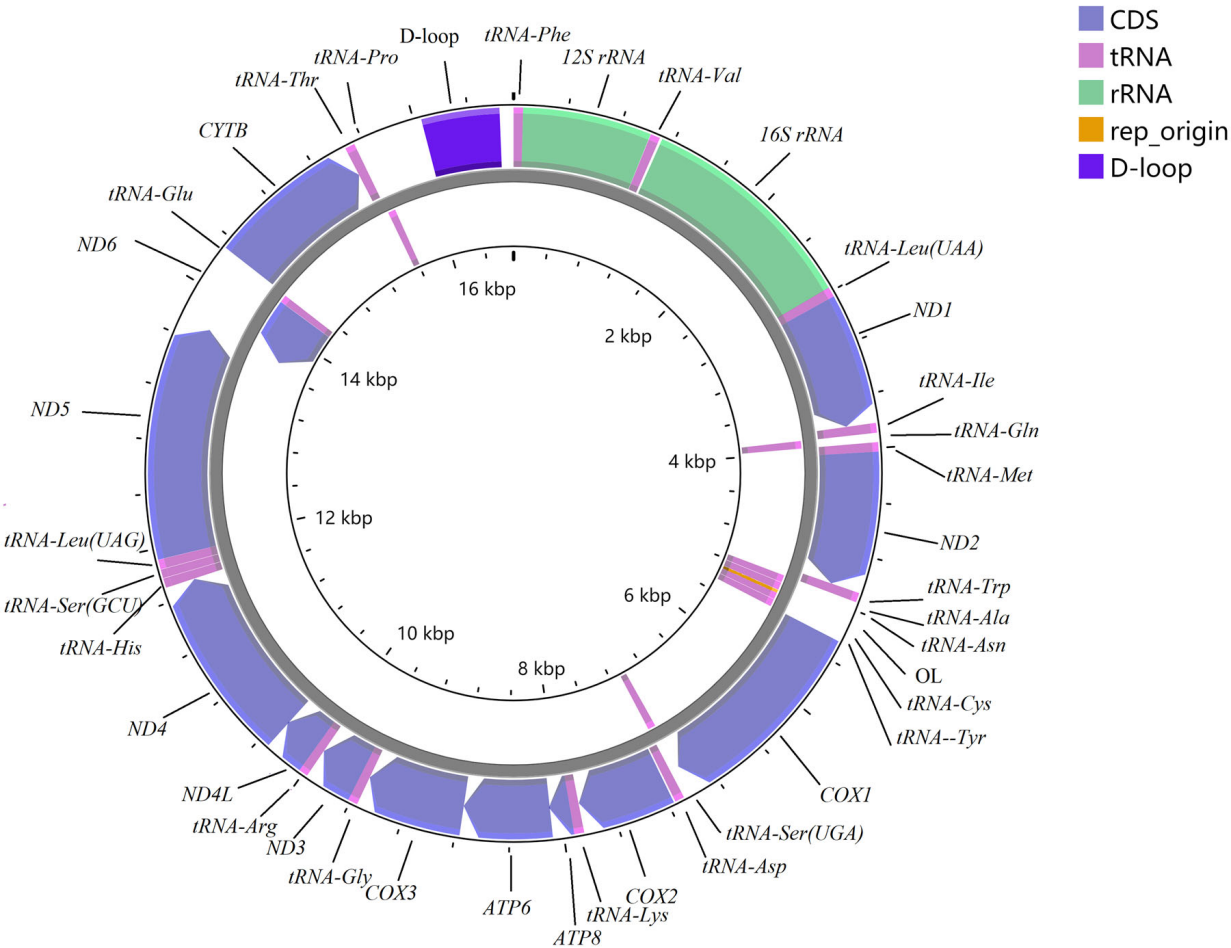
Complete mitochondrial genome map of *Devario interruptus* (GenBank: MZ853154), with 13 protein coding genes, 22 tRNAs, 2 rRNAs, and 2 non-coding regions. Genes encoded on light strand and heavy-strand were shown inner and outside of the gray circle respectively.

The complete mitogenome of *D. interruptus* was assembled to be a circular DNA molecule of 16,735 bp (T 27.9%, C 23.7%, A 33%, and G 15.4%; 60.9% AT content), that contained 13 protein-coding genes, 22 tRNAs, two rRNAs, and two non-coding regions (OL: origin of L-strand replication, 31 bp; D-loop: displacement loop region, 1125 bp). Among the 37 genes, 9 genes (*tRNA-Gln*, *tRNA-Ala*, *tRNA-Asn*, *tRNA-Cys*, *tRNA-Tyr*, *tRNA-Ser(UGA)*, *ND6*, *tRNA-Glu*, and *tRNA-Pro*) were encoded on the L-strand, the other 28 genes were encoded on H-strand. The gene composition, order, and direction were similar to the mitogenomes of species from same subfamily of Danioninae (Song et al. 2022). Characteristics of the mitochondrial genome of *Devario interruptus* was shown in Table 1 and Figure 2. The conserved 13 PCGs varied in length from 165 bp (*ATP8*) to 1,806 bp (*ND5*). Twelve PCGs have an ATG starting codon except for *COX1* that have a GTG starting codon. TAA, TAG, T, and TA were used as the termination codon. The length of 22 tRNAs ranges from 66 bp (*tRNA-Cys*) to 74 bp (*tRNA-Leu*). The length of *12S rRNA* and *16S rRNA* is 954 and 1,651 bp, respectively.

**Table 1.** Characteristics of the mitochondrial genome of *Devario interruptus.*

Gene	Start	Stop	Length(bp)	Space (+) Overlap (−)	Codons Initial/ Terminal	Strand
*tRNA-Phe*	1	70	70	0		H
*12S rRNA*	71	1024	954	2		H
*tRNA-Val*	1027	1097	71	19		H
*16S rRNA*	1117	2767	1651	−1		H
*tRNA-Leu*	2767	2840	74	0		H
*ND1*	2841	3815	975	1	ATG/TAA	H
*tRNA-Ile*	3817	3888	72	−2		H
*tRNA-Gln*	3887	3957	71	0		L
*tRNA-Met*	3958	4026	69	0		H
*ND2*	4027	5071	1045	−2	ATG/TAG	H
*tRNA-Trp*	5072	5141	70	2		H
*tRNA-Ala*	5144	5211	68	1		L
*tRNA-Asn*	5213	5285	73	2		L
OL	5288	5317	30	−1		L
*tRNA-Cys*	5317	5382	66	0		L
*tRNA-Tyr*	5383	5452	70	1		L
*COX1*	5454	7004	1551	0	GTG/TAA	H
*tRNA-Ser*	7005	7075	71	2		L
*tRNA-Asp*	7078	7147	70	12		H
*COX2*	7160	7847	688	3	ATG/T	H
*tRNA-Lys*	7851	7923	73	0		H
*ATP8*	7924	8088	165	−7	ATG/TAG	H
*ATP6*	8082	8765	684	−1	ATG/TAA	H
*COX3*	8765	9547	783	−1	ATG/TAA	H
*tRNA-Gly*	9547	9617	71	0		H
*ND3*	9618	9966	349	0	ATG/T	H
*tRNA-Arg*	9967	10034	68	0		H
*ND4L*	10035	10331	297	−7	ATG/TAA	H
*ND4*	10325	11703	1379	3	ATG/TA	H
*tRNA-His*	11707	11776	70	0		H
*tRNA-Ser*	11777	11845	69	−1		H
*tRNA-Leu*	11845	11917	73	1		H
*ND5*	11919	13724	1806	−4	ATG/TAA	H
*ND6*	13721	14242	522	2	ATG/TAA	L
*tRNA-Glu*	14245	14313	69	8		L
*CYTB*	14322	15462	1141	0	ATG/T	H
*tRNA-Thr*	15463	15535	73	5		H
*tRNA-Pro*	15541	15610	70	445		L
D-loop	16056	16632	577	103		H

OL: origin of L-strand replication; D-loop: displacement loop region

### 3.2. Phylogenetic analysis

**Figure 3.**
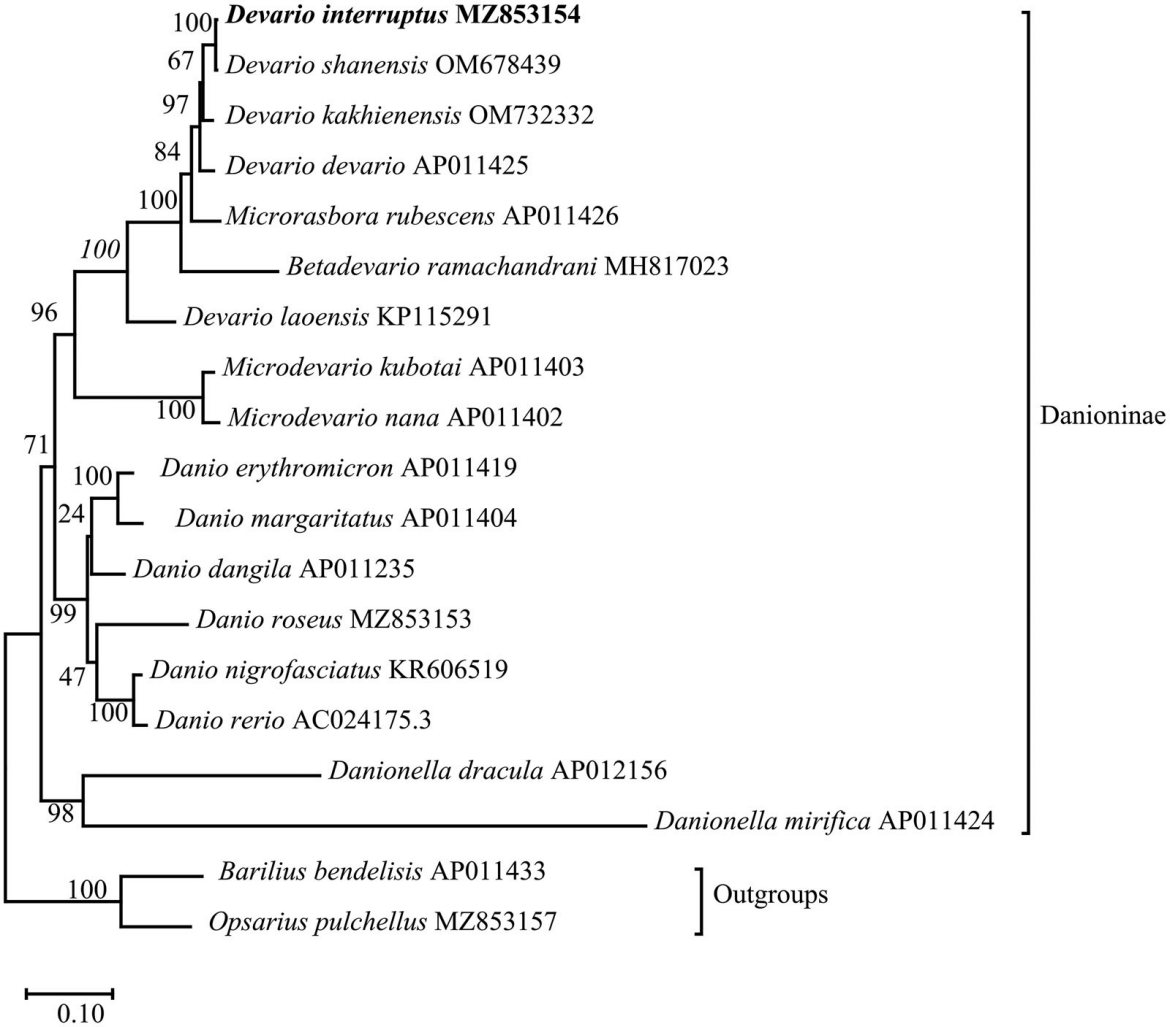
Phylogenetic reconstruction of *D. interruptus* and other 18 species based on the concatenated mitochondrial protein-coding genes using Maximum-likelihood method. *Barilius bendelisis* and *Opsarius pulchellus* were set to be outgroups. Numbers near the nodes indicated bootstrap support values from 1000 replicates.

To clarify the phylogenetic position of *D. interruptus* in subfamily Danioninae, the molecular phylogenetic tree was conducted by the Maximum-likelihood (ML) method based on 13 PCGs of *D. interruptus* and other 18 published Danionidae species from 8 genera (*Devario*, *Microrasbora*, *Betadevario*, *Microdevario*, *Danio*, *Barilius*, *Opsarius*, *Danionella*) (Broughton et al. 2001; Tang et al. 2010; Lavoué et al. 2012; Huang et al. 2016; Zhang et al. 2016; Zhang et al. 2016; Hirt et al. 2017; Norén & Kullander 2018; Kundu et al. 2019; Chen et al. 2022; Song et al. 2022), and mtREV24 + G + F (the lowest Bayesian information standard score) was selected as the optimal evolutionary model. The result of phylogenetic analysis was summarized in Figure 3. Six species of the genus *Danio* were monophyletic, and a sister group to a clade that included *D. interruptus*, *D. kakhienensis*, *D. devario*, *M. rubescens*, *B. ramachandrani*, *D. laoensis*, *M. kubotai*, and *M. nana*, and the above 15 species formed a sister group with the genus *Danionella.*

The topology revealed a close relationship between *D. interruptus* and *D. shanensis*, and *Devario* was non-monophyletic, which was inconsistent with Fang’s view that *Devario* was monophyletic, and *Danio* did not constitute a sister group with *Devario* (Fang 2003). However, the phylogenetic analysis of this current study agrees with the conclusions of Norén & Kullander (2018), that the genera *Devario*, *Microrasbora*, *Betadevario*, and *Microdevario* together constitute the sister group of *Danio*, and support the view of Tang et al. (2010) that *Danionella* was a sister group of *Danio.*

## 4. Conclusion

The complete mitochondrial genome of *Devario interruptus* was sequenced on the Illumina HiSeq platform to generate a 16,735 bp mitogenome (Genbank accession no. MZ853154). The phylogenetic position of *D. interruptus* within the subfamily of Danioninae was determined, and the results showed that *D. interruptus* was closely related to *D. shanensis*. The mitochondrial genomic data of *D. interruptus* will be essential for further genetic studies such as evolution, taxonomy, DNA barcoding, resource conservation, and phylogenetic research.

## Data Availability

The genome sequence data that support the findings of this study are openly available in GenBank of NCBI at (https://www.ncbi.nlm.nih.gov/) under the accession no. MZ853154. The associated "BioProject", "Bio-Sample" and "SRA" numbers are PRJNA769978, SAMN22187307, SRR16301567, respectively.
